# Tobacco Retail Outlets and Vulnerable Populations in Ontario, Canada

**DOI:** 10.3390/ijerph10127299

**Published:** 2013-12-17

**Authors:** Michael O. Chaiton, Graham C. Mecredy, Joanna E. Cohen, Melodie L. Tilson

**Affiliations:** 1 Ontario Tobacco Research Unit, University of Toronto, 33 Russell Street Toronto, ON, M5S 2S1, Canada; E-Mails: grahammecredy@gmail.com (G.C.M.); jocohen@jhsph.edu (J.E.C.); 2 Dalla Lana School of Public Health, University of Toronto, 33 Russell Street Toronto, ON, M5S 2S1, Canada; 3 Institute for Global Tobacco Control, Johns Hopkins Bloomberg School of Public Health, Baltimore, MD 21205, USA; 4 Non-Smokers’ Rights Association, Ottawa, ON, K1P 5G4, Canada; E-Mail: mtilson@nsra-adnf.ca

**Keywords:** tobacco, retail, availability, disparities, geospatial analysis, prevalence, school

## Abstract

Interest has been increasing in regulating the location and number of tobacco vendors as part of a comprehensive tobacco control program. The objective of this paper is to examine the distribution of tobacco outlets in a large jurisdiction, to assess: (1) whether tobacco outlets are more likely to be located in vulnerable areas; and (2) what proportion of tobacco outlets are located close to schools. Retail locations across the Province of Ontario from Ministry of Health Promotion data were linked to 2006 Census data at the neighbourhood level. There was one tobacco retail outlet for every 1,000 people over age 15 in Ontario. Density of outlets varied by public health unit, and was associated with the number of smokers. Tobacco outlets were more likely to be located in areas that had high neighbourhood deprivation, in both rural and urban areas. Outlets were less likely to be located in areas with high immigrant populations in urban areas, with the reverse being true for rural areas. Overall, 65% of tobacco retailers were located within 500 m of a school. The sale of tobacco products is ubiquitous, however, neighbourhoods with lower socio-economic status are more likely to have easier availability of tobacco products and most retailers are located within walking distance of a school. The results suggest the importance of policies to regulate the location of tobacco retail outlets.

## 1. Introduction

Tobacco is the greatest cause of death and disease worldwide, and is a risk factor for six of the eight leading causes of death in the World [1]. The World Health Organization has estimated that 100 million people have died of tobacco-related diseases during the 20th century, and that if trends continue, another 500 million may die by the end of this century [1]. 

Despite the enormous health burden, tobacco is still widely available for sale with few restrictions or requirements. Limited restrictions on availability, notably age restrictions on the sale of tobacco, have been implemented as part of many comprehensive tobacco control programs. In Canada, many provinces have restrictions on the types and locations of outlets that are permitted to sell tobacco, such as pharmacies, hospitals, and government buildings [2]. As of 2010, all provinces have restrictions on the display of tobacco products at retail. In addition, there have been many calls for reducing the availability of tobacco [3,4,5,6,7,8,9,10,11,12,13,14]. “Availability” refers to the degree of convenience experienced by consumers in obtaining tobacco products, as affected by the number, density and location of outlets, hours of sale, and in-store accessibility [6]. 

If restrictions on availability were implemented, the impact of the restrictions could have varying effects based on neighbourhood characteristics. Previous research has suggested that there is a greater density of tobacco outlets in more vulnerable neighbourhoods. In US studies examining individual cities or counties, the density of tobacco outlets is more likely to be higher in areas with lower average incomes and areas with larger African-American populations [14,15,16,17]. Others have found high densities of outlets located close to schools [18]. However, these studies are primarily limited to city or county-wide data. Peterson *et al.*, did use state-wide data to examine smoking prevalence at the county level, but did not find a relationship between outlet density and prevalence [19].

The purpose of this paper is to assess the distribution and density of tobacco outlets within an entire Canadian province, with close to 12,000 tobacco outlets, including both rural and urban areas. The specific hypotheses are as follows.

1. Increased density of tobacco retail outlets per population at the public health unit level (a unit of health administration governance larger than a municipality and approximately equivalent to a county) is associated with increased prevalence of smoking in that unit.
2. Tobacco retail outlets are more likely to be located in dissemination areas (neighbourhoods) that have lower socio-economic status, have a higher percentage of immigrants among the population and this effect will vary by urban and rural locations.
3. Tobacco retail outlets in lower socio-economic status neighbourhoods are more likely to be located close to a school and to other retailers.

## 2. Experimental Section

### 2.1. Data Sources

A list of retail outlets was obtained from the Ontario Ministry of Health and Long-Term Care (formerly Ministry of Health Promotion and Sport) Tobacco Information System database of tobacco-selling vendors. This database contains records pertaining to vendor compliance with relevant aspect of provincial law, the *Smoke Free Ontario Act*, which include prohibiting the sale of tobacco to minors, and display ban and signage provisions. The list includes the vendor address, store type, and last date visited by public health inspectors. The list was current as of June 2011.

Ground-truthing of the list was conducted in four randomly selected geographic area identified by Forward Sortation Area (the geographic area represented by the first three digits of the postal code). A data collector visited all points of sale within the area and assessed whether the store sold tobacco. Types of stores included convenience stores, gas stations, grocery stores/supermarkets, discount stores, and independent bar/restaurants. The Yellow Pages™ online directory [20] was searched to identify stores that could potentially be selling tobacco, yet were not on the Ministry list. A checklist was developed to verify whether the store sold cigarettes and to document GPS location. The data collector verified whether the store sold cigarettes by asking the clerk at the counter. A small purchase was often made to validate the data collector’s presence in the store. At each vendor visited, GPS coordinates were documented using a mobile phone device. Sensitivity of the list was extremely high at 98% (57/58 outlets on the list were selling tobacco). Specificity was 88% (57 of the 65 stores found to be selling tobacco were on the list). 

### 2.2. Mapping

The list of retail outlets obtained from the Ontario Ministry of Health and Long-Term Care was mapped via geocoding using ArcGIS 10.1 (ESRI). Each retailer’s full postal address was converted to an exact geographic location and digitally plotted on a map of Ontario. Addresses that could not be geocoded were checked for errors using Statistics Canada’s Postal Code Conversion File, as well as Canada Post’s postal code search. Remaining erroneous addresses were removed from the study. According to the list, there were 11,361 unique tobacco retailer locations in Ontario as of June 2011, of which 11,113 could be mapped. 

### 2.3. Analysis

In order to calculate retailer density at the public health unit level, retailer location data were linked to 2006 Census data using the conversion file and linked to population data. Measures of area classification type, rurality, and community size were also obtained through the postal code conversion file linkage. Area classification was broken down into three categories: Census metropolitan area (over 100,000 population), Census agglomeration (between 10,000–99,999 population), and Non-Census metropolitan or agglomeration area (under 10,000 population) [21]. Retailer location was also linked to the 2010 Canadian Community Health Survey in order to gather data on smoking rates at the public health unit level. There are 36 public health units in Ontario, corresponding to generally to the major municipalities of the province. The units are responsible for administering health promotion and disease prevention programs and support municipalities in developing and enforcing relevant public health bylaws. The public health unit is the lowest level of publically available information on smoking from the Canadian Community Health Survey. 

To measure tobacco retail availability at the neighbourhood level, tobacco retailer locations were linked to 2006 Canadian Census data to gather information on neighbourhood socio-economic status indicators. Indicators were measured at the dissemination area level (*n* = 18,922), which is a small geographic area with a population of 400 to 700 people [22], and included: neighbourhood deprivation, immigrant population, and blue-collar workers. Neighbourhood deprivation is an average score based on six variables: percentage age 25+ without high school graduation, percentage lone parent families, percentage of families receiving government transfer payments, percentage 15+ unemployed, percentage living below the low income cut off, and percentage of homes needing major repair [23]. Blue-collar workers were defined as those individuals working in trades, transport and equipment operators and related occupations, or any occupations unique to processing, manufacturing and utilities. The neighbourhood deprivation, immigrant population, and blue-collar workers variables were divided into quartiles and logistic regression was used to determine whether tobacco retailers were more likely to be found in neighbourhoods that scored high on each variable independently. 

To assess the proximity of retail outlets to schools and other retailers, circular buffers were drawn around each tobacco retailer in ArcMap, at distances of 250 m and 500 m. This enabled the measurement of a retailer’s proximity to schools or other retailers if geocoded address point of the school or other retailer point fell within this buffer. School locations were obtained from CanMap Route Logistics version 2011.3 (DMTI Spatial, Markham, ON, Canada). Analyses were conducted using SAS version 9.3 (SAS Institute Inc., Cary, NC, USA). 

## 3. Results and Discussion

### 3.1. Results

**Figure 1:**
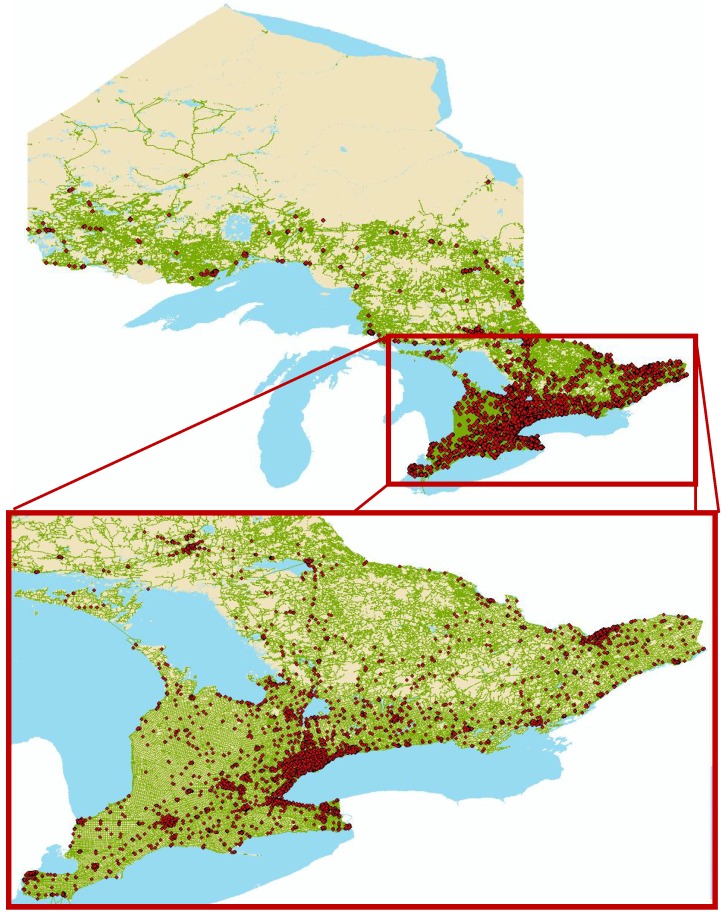
Map of tobacco retailers in Ontario, with enlarged view of southern Ontario.

See Figure 1 for the distribution of outlets across the province. Convenience stores were the most common type of outlet (56%), followed by gas stations (20%), grocery stores (9%), and bars (6%). Many retail outlets were located within walking distance of each other. In urban areas, 86% and 73% were located with 500 m and 250 m, respectively, of another retailer (data not shown). Even in rural areas, 63% and 52% of tobacco retail outlets were located with 500 m and 250 m of another retailer, respectively. 

This corresponds to approximately one location per 1,000 people aged 15+, five locations per 1,000 children <15, or five locations per 1,000 smokers. Most outlets were located in the major population centers (Table 1) with the proportion of retailers reflecting the proportion of the population; however, outlets were slightly over represented in small towns (population of less than 10,000) (16% of retailers compared to 12% of the Ontario population). 

Density of outlets per 1,000 people over 15 years old varied by the 36 public health units ranging from a low of 0.09 to a high of 1.65 with the average density of 1.05 per 1,000 people over 15 (SD (0.35 per 1,000 people over 15). Increased density was associated with higher prevalence of smoking within a public health unit (*r* = 0.36, *r*^2^ = 0.13). 

**Table 1:** Distribution of Tobacco Retail Outlets compared to the Ontario population.

	Ontario Tobacco Retailers	Ontario Population
	%	%
Area classification type *		
Census metropolitan area	74	79
Census agglomeration	10	9
Non-metropolitation/agglomeration	16	12
Rurality		
Urban	84	85
Rural	16	15
Community size		
1,500,000 +	42	42
500,000–1,499,999	10	12
100,000–499,999	23	25
10,000–99,999	9	9
<10,000	16	12

* Census metropolitan area (over 100,000 population), Census agglomeration (between 10,000–99,999 population), and Non-Census metropolitan/agglomeration (under 10,000 population).

In urban areas, tobacco retail outlets were more likely to be located in neighbourhoods that had higher deprivation, but there were fewer outlets in neighbourhoods with a high percentage of immigrants, independently of income level and neighbourhood deprivation (Table 2). There was no independent effect of the neighbourhood being predominately blue collar. In rural areas, there was similarly no correlation between retailer density and blue collar neighbourhoods, but tobacco outlets were more often located in more deprived neighbourhoods and, unlike in urban areas, neighbourhoods with higher immigrant proportion. Tobacco outlets were more likely to be found in neighbourhoods that had a higher proportion of residents receiving government assistance, being single parent families, having houses needing major repairs, and lacking a university or college degree. Unemployment rates were not associated independently with retail tobacco outlet location. 

**Table 2:** Odds of having a tobacco retail outlet by neigbourhood (dissemination area) characteristics. (*n* = 18,922 dissemination areas).

	Urban		Rural	
	Odds Ratio	*p*-*value*	Odds Ratio	*p*-*value*
Neighbourhood deprivation *				
Quartile 1—least deprivation	Referent		Referent	
Quartile 2	1.58	<0.0001	3.15	<0.0001
Quartile 3	2.04	<0.0001	3.60	<0.0001
Quartile 4—most deprivation	3.14	<0.0001	2.70	<0.0001
Immigrant population				
Quartile 1—fewest immigrants	Referent		Referent	
Quartile 2	0.99	0.77	1.61	0.0002
Quartile 3	0.88	0.01	1.52	0.2556
Quartile 4—most immigrants	0.89	0.01		
Blue-collar workers †				
Quartile 1—fewest blue-collar	Referent		Referent	
Quartile 2	1.23	<0.0001	2.387	<0.0001
Quartile 3	1.41	<0.0001	2.304	<0.0001
Quartile 4—most blue-collar	1.42	<0.0001	2.408	<0.0001

* Neighbourhood deprivation is an average score based on 6 variables: percentage 25+ without high school graduation; percentage lone parent families; percentage of families receiving government transfer payments; percentage 15+ unemployed; percentage living below the low income cut off, and percentage of homes needing major repair; † Blue-collar workers were defined as those individuals working in trades, transport and equipment operators and related occupations, or any occupations unique to processing, manufacturing and utilities.

Most retail outlets were located within walking distance of a school (Table 3). In urban areas, 68% of stores were located within 500 m of a school. In rural areas, 38% were located within 500 m of a school. Schools in lower socio-economic areas were more likely to have a retailer within walking distance (*p* < 0.001) (Table 4). For retailers with residents in the top quintile of income, 25% had schools within 250 m, and 55% had schools within 500 m. For retailers located in areas in the lowest income quintile, 33% had schools within 250 m and 73% had schools within 500 m.

**Table 3:** Number of retailers within walking distance of a school.

	Rural Retailers N (%)	Urban Retailers N (%)	Total N (%)
#of schools within 500 m of retailer			
0	1,335 (61.7%)	2,835 (31.7%)	4,170 (37.5%)
1+	828 (38.3%)	6,115 (68.3%)	6,943 (62.5%)
#of schools within 250 m of retailer			
0	1,791 (82.8%)	6,244 (70%)	8,035 (72.3%)
1+	372 (17.2%)	2,706 (30%)	3,078 (27.7%)
Total N	2,163	8,950	11,113

**Table 4:** Percent of retailers with one or more schools within walking distance (250 or 500 m), by neighbourhood income quartile (*n* = 11,013 retailers *).

Neighbourhood Income Quintiles	# of Retailers within Meters of a School N (%)	
	Retailers within 250 m	Retailers within 500 m
1 (lowest)	1,025 (33.3%)	2,236 (72.7%)
2	754 (29.2%)	1,707 (66.2%)
3	491 (23.6%)	1,159 (55.7%)
4	392 (22.3%)	950 (54.0%)
5 (highest)	385 (25.4%)	831 (54.9%)
*p*-trend	<0.0001	<0.0001

* 100 missing income variable.

### 3.2. Discussion

Availability of tobacco remains widespread across Ontario, as it does throughout most of the world. The number of retail outlets, approximately one for every thousand people older than 15, makes tobacco extremely available. Despite the tremendous burden of disease, disability, and death caused by tobacco, the widespread availability is similar to that of a normal, benign product. 

A previous Australian study using retail license data found an outlet density of two per 1,000 in New South Wales [23] while a study that used retail classification to estimate tobacco outlets found an average of 0.5 outlets per 1,000 people [24]. The density of tobacco in Ontario is slightly understated as it does not include multiple outlets per location. The number of outlets appears to have fallen in Ontario from a previous estimate of 14,500 outlets based on 2006 data [15]. Furthermore, while availability of tobacco is ubiquitous, availability is even more prevalent in vulnerable neighborhoods. Retail outlets were more likely to be present in areas that were low income. This level of increased exposure is likely to contribute to the perception that tobacco products are socially acceptable, which increases the likelihood of tobacco uptake and increases the difficulty in both making and succeeding in a quit attempt [25,26]. These findings are consistent with studies across North America that have found a relationship between socio-economic status and availability of tobacco products [27]. The relationship observed in this study is constant across rural and urban areas, suggesting that density of population, per se, is not a factor here. 

More surprising, and unlike previous studies, the relationship between immigration level in a neighborhood and tobacco retailer density differed by rurality. Consistent with American literature [14,15,16,17], rural areas with a high proportion of immigrants were more likely to have greater availability of tobacco; however, the reverse was true in urban areas. Immigrants in Canada are less likely to smoke than the general population, which may suggest that neighborhoods with more immigrants may provide an additional protective effect preventing second generation immigrants from starting to smoke [28].

Similarly, there was a modest relationship between the density of outlets and the number of smokers, with the density increasing with the prevalence of smoking within public health units. This is likely the result of two main processes: tobacco outlets follow cigarette demand, and the number of smokers is kept high by the increased supply of cigarettes. While it is impossible to distinguish these effects with this cross-sectional analysis, Novak *et al.*, found that the relationship between outlet density and population increased in magnitude with additional control for potential confounders [29]. Our analysis also uses a large unit of population size to assess smoking status, similar to or larger than the county level analysis of Peterson *et al*. [19]. Given the limitations of using public health unit as a geographic unit, any association seen in this study would likely be smaller in magnitude than if a smaller, more homogeneous geographical unit had been available to assess the relationship of smoking prevalence and outlet density. 

Curiously, there was no relationship between the blue collar status of the neighborhood and the number of outlets, in either rural or urban areas. Both low income adequacy and blue-collar status are known to be consistently associated with increased levels of smoking in Ontario [30]; however, the differentiation with respect to the presence of tobacco outlets suggests that a mechanism exists other than simple correlation between the number of smokers and the number of outlets. Research from other areas shows that higher density of fast food and alcohol outlets in vulnerable neighborhoods results in corresponding higher rates of alcohol-related problems and overweight/obesity [31,32].

Many children have availability to tobacco within a short distance of their schools. A cross-sectional analysis by Henriksen *et al.*, found that schools located in neighborhoods with a higher proportion of Hispanics and residents of lower socio-economic status were more likely to have higher tobacco retailer density [18]. Associations between availability of tobacco and smoking prevalence have also been found in Ontario [33,34]. Nearly three quarters of retailers in low income areas have a school within a 5 min walk (*i.e.*, 500 m). This proximity to tobacco retailers serves to increase tobacco exposure and the opportunity to purchase tobacco products [35,36,37] which may aid in the transition from experimenting with smoking to becoming regular smokers [34].

## 4. Limitations

Other than the cross-sectional and ecological limitations discussed in the analysis of smoking and density of outlets at the public health unit level, there are several other limitations to the interpretation of these results. The retailer outlet list, while more complete than other commercial lists, relies on entries from public health unit health inspectors, and may be an underestimate of the total number of vendors. Similarly, if there were two outlets at the same address (which could occur with a gas station and convenience store at the same location, or simply an erroneous double entry in the retailer list), only a single retailer was counted. This resulted in 1906 entries being removed from the retailer list. Consequently, our estimate represents the number of places that sell tobacco rather than the number of individual tobacco vendors. Finally, there may be systematic differences in the measurement and geocoding of addresses between urban and rural areas, as postal addresses as well as postal area units (such as dissemination areas) may be different in terms of geographic size. These findings may not be generalizable to other provinces, states or other jurisdictions as specific results should vary based on general building density, population distribution along with specific business or zoning regulations, tobacco specific or otherwise. 

## 5. Conclusions

In the province of Ontario, Canada, over 11,000 outlets sell cigarettes. This represents one outlet per 1,000 smokers, which constitutes easy availability to a highly addictive product that remains the leading cause of preventable death in Canada and the only risk factor common to the top four non-communicable diseases. A greater number of tobacco outlets means a greater likelihood that some stores will sell to underage youth but also contributes to the perception that tobacco use remains a social norm. With most tobacco outlets located within walking distance of a school and more likely to be located in deprived neighbourhoods, policy reforms related to reducing retail availability in general and retail density in these prime locations are urgently needed. More research examining tobacco outlet proximity to schools is needed to assess the impact of proximity and changes in proximity on youth tobacco use. This study was feasible because of the creation of a tobacco retailer list by the Ontario Ministry of Health and Long Term Care. Other jurisdictions should create and make available the number and location of retailers to allow research in other jurisdictions. 
